# Home Advantage in Men’s and Women’s Spanish First and Second Division Water Polo Leagues

**DOI:** 10.2478/hukin-2013-0034

**Published:** 2013-07-05

**Authors:** Jaime Prieto, Miguel-Ángel Gómez, Richard Pollard

**Affiliations:** 1 Faculty of Physical Activity and Sport Sciences, Polytechnic University of Madrid, Madrid, Spain.; 2 Department of Statistics, California Polytechnic State University, San Luis Obispo, USA.

**Keywords:** home court advantage, sex of participants, level of competition, competitive balance, water polo

## Abstract

The purpose of this study was to quantify the home advantage in both men’s and women’s First and Second Division water polo leagues, to compare the results obtained according to sex of participants and the level of competition, and to test for possible differences in home advantage when considering the interaction between these two factors. The sample comprised four seasons from 2007–2008 to 2010–2011 for a total of 1942 games analyzed. The results showed the existence of home advantage in both men’s and women’s First and Second Divisions. After controlling for the competitive balance of each league in each season, there was a significant difference between men’s and women’s leagues, with higher home advantage for men’s leagues (58.60% compared with 53.70% for women’s leagues). There was also a significant difference between the levels of competition, with greater home advantage for the Second Division (57.95% compared with 54.35% for First Division). No significant differences in home advantage were found when considering the interaction between sex of participants and the level of competition. The results in relation to sex of participants and the level of competition are consistent with previous studies in other sports such as football or handball.

## Introduction

The effect of game location (home or away) in determining the result of a sports competition is a widely studied phenomenon. It usually produces a home advantage through which athletes perform better and have more success at home than away. The main explanations that have been considered for the existence of home advantage are crowd effects, travel effects, familiarity with the field, referee or judging bias, territorial protection, playing tactics, rule changes, and psychological aspects ( Pollard, 2006 ). Even though this categorization of factors associated with the existence of home advantage is supported by the available literature, determining how they operate and interact remains a challenge ( Gómez et al., 2011 ).

The effect of home advantage has been studied in many sports competitions in different countries, both for individual sports ( Balmer et al., 2005 , in boxing; Koning, 2011 , in tennis) and for team sports ( Pollard and Gómez, 2009 , in football; Marcelino et al., 2009 , in volleyball; Gómez and Pollard, 2011 , in basketball; Prieto and Gómez, 2012 , in handball and rugby). This is especially so in Spain, where the existence of home advantage is a firmly rooted phenomenon in multiple team sports, including football, basketball, indoor soccer, handball, rugby, volleyball, roller hockey, and water polo ( Gómez et al., 2011 ). Thus, the first hypothesis predicted the existence of home advantage in Spanish water polo leagues.

The vast majority of studies on home advantage have focused on male athletes, with very few investigations comparing home advantage in men’s and women’s sports competition ( Moore and Brylinsky, 1995 ; Koning, 2005 , 2011 ; Pollard and Gómez, 2012a ). Likewise very few studies have examined the home advantage effect according to the level of competition ( Jacklin, 2005 ; Pollard, 2006 ; Sánchez et al., 2009 ) nor tested the possible differences in home advantage when considering the interaction between sex of participants and the level of competition. To the best of our knowledge, existing home advantage research in water polo is confined to a single study in men’s Spanish First Division ( Gómez et al., 2011 ), which therefore makes it impossible to know whether sex or the level of competition, or their interaction, affect home advantage in water polo. A recent reassessment by Pollard and Gómez (2012b) studied these aspects in Spanish professional handball. With regard to sex of participants they found higher values of home advantage for men (61.6%) than for women (59.2%). Therefore, the second hypothesis predicted greater home advantage for men’s leagues than for women’s leagues in Spanish water polo. According to the level of competition, Pollard and Gómez (2012b) also found significant differences, with greater home advantage values for play at Level 2 (61.3%) than at Level 1 (59.4%). Thus, the third hypothesis predicted higher home advantage values in Spanish Second Division water polo leagues. Despite these differences observed according to sex of participants and the level of competition, Pollard and Gómez (2012b) did not find significant differences when considering the interaction between these two factors. Hence, the fourth hypothesis predicted no differences in home advantage when considering the interaction between sex of participants and the level of competition in Spanish water polo leagues.

Water polo is a sport with a growing interest worldwide, particularly for research purposes, with a noticeable increase in the number of publications during recent years. The available research has tried to define the qualities of elite water polo players, and to identify the performance characteristics of the game in both men’s and women’s competitions ( Ferragut et al., 2011 ; Lupo et al., 2012 ; Escalante et al., 2012 ). Players’ qualities and performance characteristics may be related to the success of the winning teams, and therefore, to the possible advantage obtained when playing at home. However, the existing studies have not analyzed the influence of game location and quality of opposition. These variables are of great interest for coaches and players due to their importance for training plans and competition direction. Hence, studying the home advantage effect, taking into account the quality of opposition, may lead researchers to a better understanding of water polo.

Against this background the objectives of the present study were (1) to quantify the home advantage in both men’s and women’s Spanish professional First and Second Division water polo leagues, (2) to compare the results obtained according to sex of participants, (3) to compare the results according to the level of competition, and (4) to test for possible differences in home advantage when considering the interaction between sex of participants and the level of competition.

## Material and Methods

### Sample

The sample consisted of four seasons of men’s and women’s First Division and Second Division water polo leagues, from 2007–2008 to 2010–2011. All data were obtained from the open access web domain of the Spanish Swimming Federation (http://www.rfen.es). A total of 1942 games were analyzed (n= 528 in men’s First Division; n= 578 in men’s Second Division; n= 528 in women’s First Division; and n=308 in women’s Second Division).

### Quantification of Home Advantage and Competitive Balance

All the leagues under analysis had a balanced schedule of games in which each team played every other once at home and once away during the season. This league structure allows an unbiased method for quantifying the home advantage over a complete season ( Pollard and Pollard, 2005 ). Thereby, home advantage was quantified as the number of points won at home by all teams expressed as a percentage of the total number of points won at home and away ( Pollard, 2006 ), considering the point system in the analyzed seasons (3 points for a win, 1 point for a draw and 0 points for a loss). This method for calculating home advantage based on points has a potential limitation due to differences in team abilities ( Pollard and Gómez, 2009 ). When a strong team plays against a weak team, the difference in ability is often likely to make the stronger team win both at home and away and, thus, obscure the effect of home advantage. To overcome this problem a method for quantifying the competitive balance in each league and each season was introduced.

The original methodology for quantifying the competitive balance in a league ( Scully, 1989 ; Quirk and Fort, 1992 ) calculates a ratio of standard deviations (RSD) in which the actual standard deviation (ASD) of the winning percentages of the teams in a league is divided by the standard deviation that would be expected if all teams were of the same ability, referred as ideal standard deviation (ISD). RSD represents the competitive balance: the smaller the ratio the lesser the differences in team abilities and, therefore, greater competitive balance; the higher the ratio the greater the differences in team abilities and, hence, lesser competitive balance. This method was improved by Trandel and Maxcy (2011) , who realized that the calculation of ISD did not allow for home advantage, because it was based on the consideration that all the teams had the same ability, and, thus, had a probability of 0.5 (50%) to win any game against any team, either home or away. Trandel and Maxcy’s paper describes a formula that can be used to calculate a home-advantage-corrected ISD, and, therefore, a corrected measure of competitive balance. Their methodology was used in the present study.

### Statistical Analysis

To test for differences between men’s and women’s Spanish professional First and Second Division water polo leagues a two-way analysis of variance (ANOVA) was performed, with home advantage as the dependent variable, sex of participants and the level of competition as the fixed factors, and with competitive balance as a covariate. Effect size was measured by Cohen’s f index ( Cohen, 1977 ). Statistical significance was set at 5% and all analyses were performed using the statistical package software PASW Statistic (IBM SPSS Statistics, Chicago, US).

## Results

Table 1 shows home advantage and competitive balance for each season in each league under analysis. With regards to the first objective, the results showed the existence of home advantage in both men’s and women’s First and Second Division leagues, with values above 50% in all the cases studied.

After controlling for the competitive balance of each league in each season, the statistical analysis showed the following results in relation to the stated objectives. With respect to the sex of participants (second objective), the results showed a significant difference in home advantage between men’s and women’s leagues, with higher values for men’s leagues (58.60% compared with 53.70%; F1,11=15.49; p=0.002; f=0.85). With regards to the level of competition (third objective), the results showed a significant difference in home advantage between the First and Second Divisions, with greater home advantage for the Second Divisions: 57.95% compared with 54.35% (F1,11=8.75; p=0.013; f=0.56). Finally, regarding the fourth objective, the results showed no significant differences in home advantage when considering the interaction between sex of participants and the level of competition (i.e. sex of participants * level of competition) (F1,11=0.00; p=0.980; f=0.001).

## Discussion

The purpose of this study was to quantify the home advantage in both men’s and women’s Spanish professional First and Second Division water polo leagues, to compare the results obtained according to sex of participants and the level of competition, and to test for possible differences in home advantage when considering the interaction between these two factors.

The first hypothesis was supported. The results showed the existence of home advantage in both men’s and women’s First and Second Division leagues, with values above 50% in all the leagues. With respect to men’s water polo leagues, the existence of home advantage is in accordance with a recent study by Gómez et al. (2011) , which supported the existence of a home advantange effect in nine different men’s professional team sports in Spain, including water polo. More generally, although water polo was not included, this effect in men’s leagues is consistent with the results of a meta-analysis conducted by Jamieson (2010) , in which a significant advantage for home teams in men’s competitions was observed for multiple sports across all conditions, with time era, season length, game type, and sport moderating the effect. With respect to women’s water polo leagues, the existence of home advantage is consistent with several other studies which reported significant home advantage values in women’s competitions ( Gómez et al., 2007 , in the Spanish professional basketball league; Baghurst and Ford, 2008 , in collegiate gymnastics teams in the United States; Pledger and Morton, 2010 , in national netball competitions in Australia, New Zealand and England).

With respect to the second hypothesis, the results confirmed the prediction of greater home advantage values for men’s leagues than for women’s leagues. This is consistent with the available literature in other sports, which shows evidence of a greater home advantage in men’s competitions than in women’s competitions. However, very little research has been published in this respect. To our knowledge no previous studies have been published in water polo. In football, a study conducted by Pollard and Gómez (2012a) across 26 European countries, showed greater home advantage values in men’s leagues in every country under analysis. In tennis, Koning (2011) found that although significant home advantage exists for men, the performance of women tennis players appears to be unaffected by home advantage. These differences can be explained from two main perspectives: according to gender theories, or according to some of the factors associated with the existence of home advantage.

On the one hand, according to gender theories, existing research findings suggest that sex differences in teams’ and players’ performance may be related to the anthropometric, physical, cognitive, motor, and physiological differences between women and men and their effect on performance profiles ( Sampaio et al., 2004 ; João et al., 2010 ). For example in football, some studies argue that females’ performance decreases due to their inferior athletic, technical, and tactical football ability conditions ( Konstadinidou and Tsigilis, 2005 ; Chalabaev et al., 2008 ). In handball, a study by Zapartidis et al. (2011) on the motor abilities of young male and female players, showed that in the older age groups (13–13.9, 14–14.9 and 15–15.9 years), besides having the same performance demands and training experience, males performed better than females in motor abilities that are important for handball, with sport-specific training appearing to be not sufficient to attenuate these sex differences in motor performance. Moreover, in relation to psychological factors, an interesting recent research in professional tennis relative to performance feedback ( Wozniak, 2012 ), found that men exhibit a “hot hand” effect after doing well in a tournament than can last for several further tournaments, while females are affected by only their performance in their last tournament.

On the other hand, the greater home advantage in men’s competitions can also be explained according to some of the factors associated with the existence of home advantage, such as crowd effects and referee bias, territorial protection, and psychological aspects. Regarding the crowd effects, the traditionally higher match attendance for men’s competitions compared with women’s is likely to play a role in the greater men’s home advantage ( Pollard and Gómez, 2012a ). **However, and despite what the fans think, recent studies have provided increasing evidence that the primary effect of the crowd is not to give an advantage to the home team or a disadvantage to the away team ( Pollard, 2008a ), but to influence referee decisions in favour of the home team, and, therefore, establish a referee bias ( Nevill et al., 2002 ; Dawson et al., 2007 ).** Regarding the factor of territorial protection, testosterone levels have been associated with a variety of athletic qualities, including aggressive behavior. In a study in football, Wolfson et al. (2007) showed that testosterone levels in males before a match were greater when performing at home than away, but that the corresponding difference for females was much less. They theorized that since testosterone level is associated with more aggressive behavior, this, combined with a greater sense of territorial protection might partially explain the home advantage being greater for males than females. Furthermore, certain psychological aspects such as an increased susceptibility to environmental changes experienced by women players may influence women’s reduced home advantage ( João et al., 2010 ).

With regard to the level of competition, the results confirmed the third hypothesis, with greater home advantage values for the Second Divisions than for the First Divisions. No relevant previous studies have been published in water polo. In other sports, the home advantage differences by the level of competition are inconsistent with no clear pattern. In football, some studies have found home advantage in the Second Divisions greater than in the First Divisions ( Jacklin, 2005 ; Silva et al., 2008 ; Pollard and Gómez, 2012b ), while others have failed to show any substantial differences between the top two levels ( Pollard, 2006 ; Dosseville, 2007 ; Seckin and Pollard, 2008 ; Sánchez et al., 2009 ). Interestingly, no study has claimed that home advantage at level 1 is higher than at level 2, a situation that would be expected if crowd size was a factor in affecting home advantage. Finally, the results supported the fourth hypothesis which predicted no significant differences in home advantage when considering the interaction between sex of participants and the level of competition, and, therefore, suggested that the influence of these two factors on home advantage acts independently. To the best of our knowledge, the only previous study to consider this interaction was performed in handball ( Pollard and Gómez, 2012b ) and also failed to find any interaction effect on home advantage between sex of participants and the level of competition.

Regarding practical implications, existing studies argue that there is relatively little that the home teams can do to increase their advantage, but definite steps can be taken by visiting teams to minimize the away disadvantage. Specific routines can be incorporated by coaches and team psychologists as part of game preparation, as well as during the game itself ( Wolfson and Neave, 2004 ; Pollard, 2008b ). To establish a set routine for away games, to give players as much information as possible about each away venue, to train discipline by focusing on the game and not getting distracted by noise and abuse from the home crowd by which may generate negative feelings and thereby, poor functional assertive behaviours, and to avoid protest against referee decisions are some of the aspects on which coaches, players and team psychologists can work in order to minimize the disadvantage of playing away from home.

In conclusion, the results of the study showed the existence of home advantage in men’s and women’s Spanish First and Second Division water polo leagues. Home advantage was greater for men than for women. It was also greater for the Second Division than for the First Division. There were no significant differences in home advantage when considering the interaction between sex of participants and the level of competition. The results lend support to the universality of the existence of home advantage in all sporting competitions. The phenomenon is now confirmed for yet another sport, water polo. Since the study was confined to four leagues in a single country, and since there is a paucity of home advantage research taking into account sex and the level of competition, future work should attempt to replicate the results in other countries both for water polo and for other sports. Thus, this study provides new insights for further understanding of the home advantage effect in general, and the game of water polo in particular.

## Figures and Tables

**Table 1:** *
Home advantage (HA) and competitive balance (CB) in men’s and women’s Spanish First and Second Division water polo leagues (2007–2008 to 2010–2011)
*

League Gender	League Division	No. of Games	2007–2008		2008–2009		2009–2010		2010–2011		HA all seasons	
			HA(%)	CB	HA(%)	CB	HA(%)	CB	HA(%)	CB	* M *	* SD *
Men’s	First	528	53.11	2.82	58.51	2.44	57.22	2.82	57.40	2.43	56.59	2.38
Men’s	Second	578	59.85	2.81	63.21	2.45	57.77	2.85	61.60	2.33	60.61	2.34
Women’s	First	528	52.69	2.40	50.77	2.39	51.14	2.93	53.83	2.16	52.11	1.42
Women’s	Second	308	50.91	2.44	61.21	2.43	55.17	2.96	53.90	2.66	55.30	4.33
